# Predict cognitive decline with clinical markers in Parkinson’s disease (PRECODE-1)

**DOI:** 10.1007/s00702-019-02125-6

**Published:** 2019-12-18

**Authors:** Heather Wilson, Gennaro Pagano, Tayyabah Yousaf, Sotirios Polychronis, Rosa De Micco, Beniamino Giordano, Flavia Niccolini, Marios Politis

**Affiliations:** Neurodegeneration Imaging Group, Maurice Wohl Clinical Neuroscience Institute, Institute of Psychiatry, Psychology and Neuroscience (IoPPN), King’s College London, 125 Coldharbour Lane, Camberwell, London, SE5 9NU UK

**Keywords:** Parkinson’s disease, Cognitive decline, Cognitive impairment, Predictors

## Abstract

Over the course of the disease, about 80% of Parkinson’s disease patients will develop cognitive impairment. However, predictive factors associated with cognitive decline are still under investigation. Here, we investigated which clinically available markers are predictive of cognitive impairment in a cohort of early drug-naïve Parkinson’s disease patients. 294 drug-naïve Parkinson’s disease patients, who were cognitively normal at baseline, were recruited from the Parkinson’s Progression Markers Initiative. At 36-month follow-up, patients were diagnosed with cognitive impairment according to two levels: Level 1 diagnosis was defined as MoCA < 26 and Level 2 diagnosis was defined as MoCA < 26, alongside an impaired score on at least two neuropsychological tests. Predictive variables with a validated cut-off were divided into normal or abnormal measures, whilst others were divided into normal or abnormal measures based on the decile with the highest power of prediction. At 3 years’ follow-up, 122/294 Parkinson’s disease (41.5%) patients had cognitive decline. We found that age at Parkinson’s disease onset, MDS-UPDRS Part-III, Hopkin’s Learning Verbal Test-Revised Recall, Semantic Fluency Test and Symbol Digit Modalities Test were all predictors of cognitive decline. Specifically, age at Parkinson’s disease onset, Semantic Fluency Test and symbol Digit Modalities Test were predictors of cognitive decline defined by Level 2. The combination of three abnormal tests, identified as the most significant predictors of cognitive decline, gave a 63.6–86.7% risk of developing cognitive impairment defined by Level 2 and Level 1 criteria, respectively, at 36-month follow-up. Our findings show that these clinically available measures encompass the ability to identify drug-naïve Parkinson’s disease patients with the highest risk of developing cognitive impairment at the earliest stages. Therefore, by implementing this in a clinical setting, we can better monitor and manage patients who are at risk of cognitive decline.

## Introduction

Cognitive impairment (CI) is currently considered to be one of the most common non-motor aspects of Parkinson’s disease (PD), which greatly affects quality of life (Schrag et al. 2017), increases caregiver burden and nursing home placement (Aarsland et al. 2005). Compared to the general population, the presence of PD increases the risk of developing dementia by six times, with approximately 80% of PD patients going onto develop dementia over the course of the disease (Aarsland et al. 2003). CI in PD appears to be common, even at the time of PD diagnosis, occurring in 20–50% of PD patients (Aarsland et al. 2005). Despite the impact of CI in PD, clinical predictive factors which are associated with cognitive decline are still under investigation and validation for use in clinical practice to identify early drug-naïve PD patients at higher risk of cognitive decline.

Several clinical risk factors of cognitive decline in PD have been identified including older age of onset, greater motor symptom burden, having an akinetic-rigid subtype and olfactory dysfunction (Bohnen et al. 2010; Baba et al. 2012). However, these predictors were not consistent across the studies (Mollenhauer et al. 2006; Compta et al. 2009; Alves et al. 2010). The Cambridgeshire Parkinson’s Incidence from GP to Neurologist (CamPaIGN) study, which is the largest study conducted on PD patients to date, reported that one third of PD patients had CI at time of the diagnosis (Foltynie et al. 2004). At 3–5 years’ follow-up, 10% of the patients had developed dementia, with the prevalence of CI rising up to 57% (Williams-Gray et al*.* 2009). By 10 years, the prevalence of dementia was 46% (Williams-Gray et al. 2013). The most imperative neuropsychological predictors of cognitive decline, in this study, included semantic fluency and the ability to copy an intersecting pentagons figure. Recently, Liu and colleagues presented a clinical-genetic score to predict global cognitive impairment within 10 years of PD onset based on clinical variables and the β-glucocerebrosidase (GBA) genotype (Liu et al. 2017).

Here, we have used data collected from the Parkinson’s Progression Marker Initiative (PPMI) to investigate clinically available predictors of cognitive decline in drug-naïve PD and to identify a subgroup of PD patients who are at higher risk of cognitive decline.

## Methods

### Participants and clinical characteristics

Data were obtained from the PPMI database (www.ppmi-info.org/data), which is an ongoing observational multicentre cohort study of PD patients. From a total 412 PD patients, we identified and included 294 PD patients who were not cognitively impaired at baseline (MoCA ≥ 26) and had a complete 36-month follow-up. All PD patients underwent an initial screening visit followed by a baseline visit where demographic and clinical information were collected (Table 1). Motor, non-motor, cognitive and neuropsychological assessments were also collected at baseline and follow-up visits over a 36-month period.

**Table 1:** Demographic and clinical characteristics of Parkinson’s disease patients who develop cognitive impairment (Level 1) at 3-year follow-up

	All PD (*n* = 294)	Cognitive impairment (*n* = 122)	No cognitive impairment (*n* = 172)
Age^a^	60.81 ± 9.76	64.47 ± 8.74	58.22 ± 9.63*
Age of onset^a^	60.26 ± 9.72	63.87 ± 8.78	57.70 ± 9.55*
Gender male, % (*n*)	65.3% (192)	76.2% (93)	57.6% (99)*
Disease duration (months)^b^	6.64 ± 6.71	7.16 ± 7.04	6.27 ± 6.46
Year of education^b^	15.72 ± 2.96	15.61 ± 3.42	15.80 ± 2.60
Family history of PD, % (*n*)	25.2% (74)	23% (28)	26.7% (46)
H&Y stage^b^	1.48 ± 0.50	1.55 ± 0.5	1.43 ± 0.5*
Akinetic-rigid subtype, % (*n*)	59.9% (176)	54.9% (67)	63.4% (109)
MDS-UPDRS II^b^	5.84 ± 4.25	6.88 ± 4.32	5.11 ± 4.06*
MDS-UPDRS III^a^	20.04 ± 8.48	22.69 ± 8.98	18.17 ± 7.61*
MDS-UPDRS I^b^	1.24 ± 1.63	1.49 ± 1.90	1.06 ± 1.40*
MDS-UPDRS I Questionnaire^b^	4.12 ± 3.06	4.76 ± 3.10	3.66 ± 2.96*
GDS^b^	2.28 ± 2.47	2.72 ± 2.59	1.96 ± 2.34*
SCOPA-AUT^a^	9.50 ± 6.35	10.76 ± 6.56	8.59 ± 6.05*
ESS^b^	5.77 ± 3.41	6.07 ± 3.59	5.56 ± 3.28
RBDQ^b^	4.02 ± 2.60	4.45 ± 2.89	3.72 ± 2.34
UPSIT^a^	22.62 ± 8.15	20.68 ± 8.51	23.99 ± 7.61*
MoCA^b^	28.06 ± 1.33	27.51 ± 1.22	28.46 ± 1.26*
Letter–Number Sequencing Test^a^	14.59 ± 2.02	14.20 ± 1.79	14.66 ± 2.07
Semantic Fluency Test^a^	49.92 ± 11.32	45.48 ± 10.82	53.09 ± 10.61*
HVLT-R recall^a^	47.23 ± 10.46	43.30 ± 9.49	50.02 ± 10.24*
HVLT-R recognition discrimination^a^	49.41 ± 11.36	48.55 ± 13.77	50.02 ± 9.29
Symbol Digit Modalities Test^a^	42.33 ± 9.39	38.11 ± 9.46	45.33 ± 8.11*
Benton judgement of line orientation^b^	13.03 ± 1.98	12.56 ± 2.20	13.37 ± 1.74*

Data shown as mean ± SD

*EES* Epworth Sleepiness Scale, *GDS* 15-item Geriatric Depression Scale, *H&Y* Hoehn and Yahr, *HVLT-R* Hopkins Verbal Learning Test-Revised, *MDS-UPDRS* Movement Disorder Society sponsored Unified Parkinson Disease Rating Scale, *MoCA* Montreal Cognitive Assessment Scale, *PD* Parkinson’s disease, *RBDQ* REM sleep behaviour disorders (RBD) screening questionnaire, *SCOPA-AUT* Scales for Outcomes in Parkinson’s disease—autonomic, *UPSIT* University of Pennsylvania Smell Identification Test

^*^P values < 0.05 (^1^*t *tests and ^2^Mann–Whitney *U* tests)

Institutional Boards of all participating sites have approved the study, and all subjects have provided written informed consent. All PD patients were recruited between 2010 and 2015, diagnosed with PD less than 2 years prior to a screening visit, never treated with dopaminergic supplementation and presented with two among bradykinesia, resting tremor and rigidity or with asymmetric resting tremor/bradykinesia at screening. The diagnosis was confirmed by the presence of dopaminergic deficit at [^123^I]FP-CIT SPECT imaging (Qamhawi et al. 2015).

Demographic information of PD patients was obtained including age, sex, years of education, age of disease onset, date of diagnosis, family history of PD and presenting motor features. Disease stage and severity were assessed using Hoehn & Yahr (H&Y) scale and Movement Disorder Society sponsored Unified Parkinson Disease Rating Scale (MDS-UPDRS). Motor burden was assessed using the MDS-UPDRS Part-III and -II. Global non-motor symptoms burden was assessed with MDS-UPDRS Part-I and MDS-UPDRS Part-I self-administered patient questionnaire. Non-motor features were evaluated using specific assessments for: depression, assessed with the short version of the Geriatric Depression Scale (15-item); autonomic dysfunction, assessed with the Scales for Outcomes in Parkinson’s disease-Autonomic (SCOPA-AUT); excessive daytime sleepiness (EDS), evaluated with the Epworth Sleepiness Scale (ESS); REM Sleep Behaviour Disorders (RBD), evaluated with the RBD screening questionnaire (RBDSQ) and olfactory dysfunction, measured with the University of Pennsylvania Smell Identification Test (UPSIT). Cognitive function was assessed using the Montreal Cognitive Assessment (MoCA) and using six neuropsychiatric assessments: Letter–Number Sequencing Test, Semantic Fluency Test (three-word categories in 60 s trials), Hopkin’s Learning Verbal Test-Revised (HVLT-R) Recall, HVLT-R Recognition Discrimination, Symbol Digit Modalities Test and Benton Judgement of Line Orientation.

### Primary endpoint

Cognitive decline was the primary outcome of the study and was established by the study physicians at the follow-up visits. Visits took place in the outpatient unit of the reference hospitals once every 12 months. The follow-up period was terminated at the 36-month follow-up visit if they had not developed CI or at the follow-up visit at which the patients had developed CI. Cognitive decline was classified according to the MDS PD-MCI criteria using two levels: Level 1 criteria primarily for use in a clinical setting and a more stringent Level 2 criteria primarily for use in a research setting (Litvan et al. 2012). Level 1 diagnosis included all PD patients who had a MoCA score < 26; Level 2 diagnosis included all PD patients with Level 1 diagnosis, who subjectively complained of cognitive issues, and had at least 2 neuropsychological test scores (of HVLT Total Recall, HVLT Recognition Discrimination, Benton Judgement of Line Orientation, Letter–Number Sequencing, Semantic Fluency Test and/or Symbol Digit Modalities; irrespective of test domain) greater than 1.5 standard deviation below the age and education-standardized mean score based on published standards in healthy controls (Weintraub et al. 2015).

### Statistical analysis

Statistical analysis was performed using the Statistical Package for Social Sciences (SPSS 22.0) software (SPSS Inc., Chicago, Illinois). For all variables, variance homogeneity and Gaussianity were tested using the Kolmogorov–Smirnov test. Continuous variables are expressed as mean ± standard deviation and compared with *t test*, if normally distributed, and with the Mann–Whitney *U* test, if not normally distributed. Categorical variables are expressed as proportions and compared using a *χ*^2^ test. To determine the independent predictors of cognitive decline, multivariate Cox proportional hazards analyses (backward elimination regression for Level 1 and Level 2 criteria) were performed including all demographic, clinical and neuropsychological measures. Only the time to occurrence of the first event was used in the Cox model. To identify a PD sub-phenotype at higher risk of CI, the predictors with the highest Wald scores were used to divide the patients in subgroups. Kaplan–Meier estimates and curves were generated, and comparisons were made using the log-rank (Mantel–Cox) test. *P* < 0.05 was considered statistically significant.

## Results

### Characteristics of PD patients

We studied 294 PD patients, with mean disease duration 6.6 ± 6.7 months (Table 1). At 3-year follow-up, 122/294 PD (41.5%) patients had a cognitive decline at Level 1, of which 53/122 (43.4%) received a confirmed diagnosis at Level 2. Patients who went onto develop cognitive decline were significantly older, had worse motor symptoms, were more depressed, exhibited more severe autonomic dysfunction, had elevated anosmia and had worse performance across several neuropsychological tests (*P* < 0.05) (Table 1).

### Predictors of cognitive decline

Backward elimination regression analysis for cognitive decline defined by Level 1 revealed the following predictors: age of onset [hazard ratio (HR) 1.03, 95% confidence interval (CI) 1.01–1.06; Wald 6.75 *P* = 0.009], MDS-UPDRS Part-III (HR 1.03, 95% CI 1.00–1.05; Wald 5.20; *P* = 0.023), Semantic Fluency Test (HR 0.98, 95% CI 0.96–1.00; Wald 4.03; *P* = 0.045), Symbol Digit Modalities Test (HR 0.98, 95% CI 0.95–0.99; Wald 4.18; *P* = 0.041), Hopkin’s Learning Verbal Test-Revised Recall (HR 0.972, 95% CI 0.954–0.991; Wald: 8.18 *P* = 0.004) and MoCA (HR 0.81, 95% CI 0.70–0.95; Wald 7.00; *P* = 0.008).

Backward elimination regression analysis for cognitive decline defined by Level 2 indicated the following predictors (Table 2): age (HR 0.48, 95% CI 0.24–0.98; Wald 4.12; *P* = 0.042), age of onset (HR 2.24, 95% CI 1.09–4.58; Wald 4.84; *P* = 0.028), MDS-UPDRS Part-III (HR 1.06, 95% CI 1.02–1.10; Wald 7.36; *P* = 0.007), Semantic Fluency Test (HR 0.95, 95% CI 0.92–0.99; Wald 8.12; *P* = 0.004), Symbol Digit Modalities Test (HR 0.93, 95% CI 0.89–0.97; Wald 12.97; *P* = 0.0003) and Benton Judgement of Line Orientation (HR 0.82, 95% CI 0.71–0.94; Wald 7.96; *P* = 0.005).

**Table 2:** Multivariate Cox hazard analysis for cognitive decline

	HR	95.0% CI for HR		*P* value	Wald
		Lower	Upper		
Level 1					
Age at onset	1.03	1.01	1.06	0.009	6.75
MDS-UPDRS Part-III	1.03	1.00	1.05	0.023	5.20
Semantic Fluency Test	0.98	0.96	1.00	0.045	4.03
Symbol Digit Modalities Test	0.98	0.95	0.99	0.041	4.18
HVLT-R recall	0.97	0.95	0.99	0.004	8.18
MoCA	0.81	0.70	0.95	0.008	7.00
Level 2					
Age	0.48	0.24	0.98	0.042	4.12
Age at onset	2.24	1.09	4.58	0.028	4.84
MDS-UPDRS Part-III	1.06	1.02	1.10	0.007	7.36
Semantic Fluency Test	0.95	0.92	0.99	0.004	8.12
Symbol Digit Modalities Test	0.93	0.89	0.97	0.0003	12.97
Benton judgement of line orientation	0.82	0.71	0.94	0.005	7.96

PD patients were then divided into subgroups according to the three most significant predictors of cognitive decline, as determined by the Wald scores. For Level 1, these included: Hopkin’s Learning Verbal Test-Revised Recall, age of onset and MDS-UPDRS Part-III; and for Level 2, these included: Symbol Digit Modalities Test, Semantic Fluency Test and age of onset. Age of onset and MDS-UPDRS Part-III were divided in deciles and subgrouping was based on the decile with the highest power of prediction. We found that the best cut-off for age of onset was 60 years of age and for MDS-UPDRS Part-III was 20. A cut-off value of 35 was ascertained for Semantic Fluency Test, Symbol Digit Modalities Test and Hopkin’s Learning Verbal Test-Revised Recall as it was greater than 1.5 standard deviation below the age and education-standardized mean score based on published standards in healthy controls. The cut-off values for Benton Judgement of Line Orientation and MoCA was 17 and 26, respectively. However, there were no patients who had a score below these cut-offs, thus these variables were excluded from the grouping process.

**Fig. 1:**
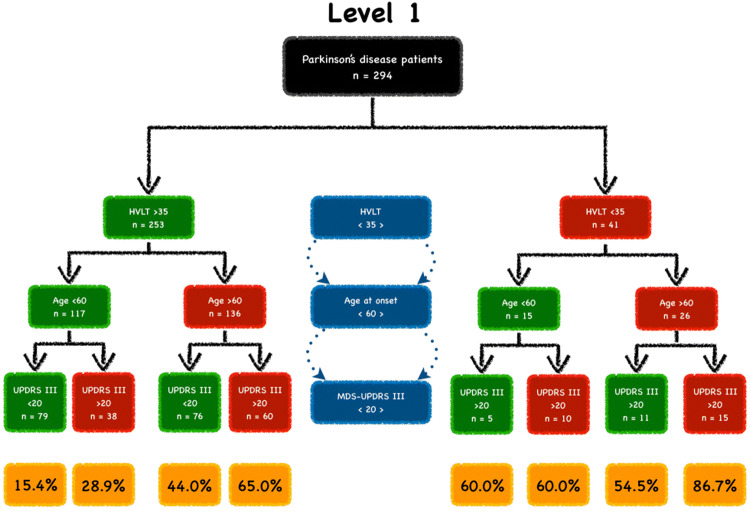
Parkinson’s disease (PD) sub-phenotypes at different risk of cognitive impairment (CI) as defined by Level 1. PD patients were grouped by Hopkin’s Learning Verbal Test-Revised Recall (HVLT), age at onset, and MDS-UPDRS III. Green boxes indicate normal variables and red boxes indicate abnormal boxes. The percentages of CI development (yellow boxes) were shown at the end of the study (36-month follow-up)

**Fig. 2:**
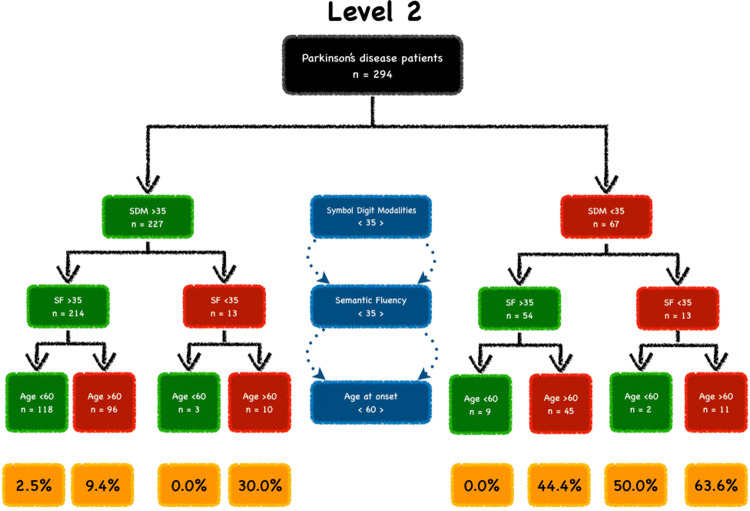
Parkinson’s disease (PD) sub-phenotypes at different risk of cognitive impairment (CI) as defined by Level 2. PD patients were grouped by Semantic Fluency (SF), Symbol Digit Modalities (SDM) and age at onset. Green boxes indicate normal variables and red boxes indicate abnormal boxes. The percentages of CI development (yellow boxes) were shown at the end of the study (36-month follow-up)

**Fig. 3:**
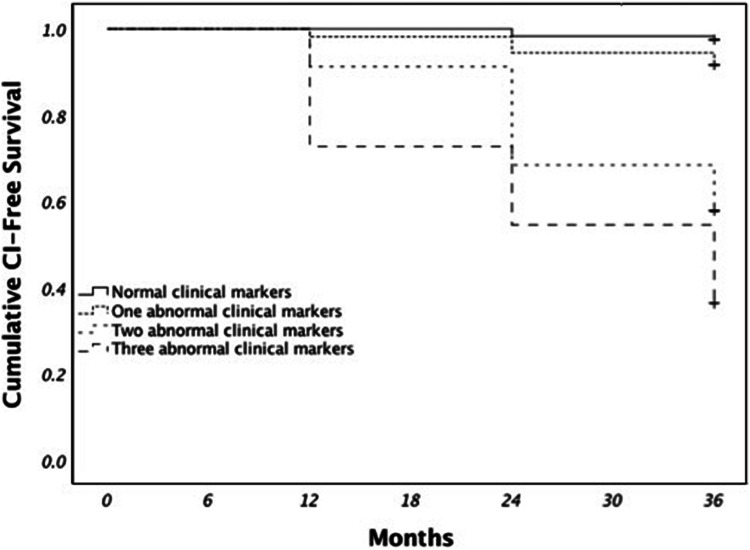
Kaplan–Meier overall survival curves for the development of cognitive impairment (CI), as defined by Level 2 diagnostic criteria, at 36-month follow-up for Parkinson’s disease (PD) patients with normal clinical markers, and with one, two or three abnormal clinical markers. Log Rank (Mantel–Cox) = 42.92 *P* < 0.0001

PD patients with a Hopkin’s Learning Verbal Test-Revised Recall Test score < 35, age at onset > 60 and MDS-UPDRS Part-III > 20 had an 86.7% risk of developing CI, as defined by Level 1, at 36-month follow-up (Fig. 1). PD patients who scored < 35 on the Symbol Digit Test and Semantic Fluency Test and had an age at onset > 60 years had a 63.6% risk of developing CI, as defined by Level 2, at 36-month follow-up (Fig. 2). Compared with PD patients with normal clinical markers, PD patients with one, two or three abnormal clinical markers had higher risk of CI, as defined by Level 2, at 36-month follow-up (Fig. 3).

## Discussion

Our findings demonstrate that the combination of three clinically available measures could identify 64% to 87% of drug-naïve PD patients that will develop CI, defined by Level 2 and Level 1 criteria, respectively, over 36-month follow-up among PD patients who were cognitively normal at the time of PD diagnosis. Specifically, increased age at PD onset, increased motor symptom severity as assessed with the MDS-UPDRS Part-III, and poor performance on Hopkin’s Verbal Learning Test-Revised Recall Test were predictors of cognitive decline defined by Level 1. While, increased age at Parkinson’s disease onset and, poor performance Sematic Fluency Test and Symbol Digit Modalities Test identified of PD patients with increased risk for cognitive decline defined by the more stringent Level 2 criteria over a 36-month period. Developing and validating models with the power to predict cognitive decline in PD patients from the earliest stages of the disease is critical to aid the development of targeted interventions to prevent the progression of cognitive decline and to provide a feasible tool for clinicians to better monitor patients for cognitive decline.

Age of onset, MDS-UPDRS Part-III, Semantic Fluency, Symbol Digit Modalities and Hopkin’s Verbal Learning Tests are easily administrable within a clinical setting as they require minimal time and are inexpensive assessments. Our findings corroborate with previous reports from the CamPaIGN study, which demonstrated that PD patients with a good outcome over 10 years, including cognitive performance, had significantly lower MDS-UPDRS Part-III scores and unimpaired semantic fluency (Williams-Gray et al. 2013). Our findings are also in line with the Oxford Discovery cohort that comprised of 155 patients who were followed for a period of 18 months (Hu et al. 2014). The authors of this study showed that older age and more advanced H&Y motor staging were predictors of lower MoCA scores, though the gold standard of CI and dementia clinical diagnosis was not employed in this study (Litvan et al. 2012; Hu et al. 2014). The novelty of our study lies in the fact that the clinical assessments that hold a predictive power for CI were performed on PD patients at the drug-naïve stage, thus enabling clinicians to monitor patients for cognitive decline and introduce therapeutic interventions at the earliest stages. Moreover, compared to other studies that assessed the prospective development of CI in PD (Weintraub et al. 2015; Schrag et al. 2017), we ensured that PD patients who were cognitively normal at baseline and had a complete 36-month follow-up were included in our study.

Whilst Schrag and colleagues similarly used the PPMI cohort to identify biomarkers and clinical variables that had predictive value for cognitive impairment, these researchers evaluated an amalgamation of both clinical and non-clinical variables over a 24-month period, assessing cognitive decline using a change in MoCA scores (Schrag et al. 2017). They reported that CSF Aβ42, caudate dopamine transporter (DAT) uptake, age, RBDQ and UPSIT were all associated with CI. Similarly, in a study carried out by our group exploring the association between cognitive decline and microstructural changes in the cholinergic system, we found that degeneration of the nucleus basalis of Meynert predicted the development of CI (Schulz et al. 2018). However, given that we focused on using simple, clinically available measures, which could be employed in a clinical setting to monitor PD patients, we only included clinical assessments that are easy and straightforward to implement, thus we did not include CSF markers or quantitative neuroimaging techniques. Our group recently demonstrated that PD patients with a combination of reduced CSF Aβ42, increased CSF total tau and reduced caudate DAT uptake had a 65% risk of developing CI, defined by Level 2 criteria, at 36-month follow-up (Yousaf et al. 2019). The predictive value of abnormal caudate DAT [^123^I]FP-CIT SPECT alone was only 10% (Yousaf et al. 2019). The identification of predictive biological and neuroimaging variables provides important insights into a possible pathophysiological link between CSF Aβ42, total tau and striatal dopaminergic integrity with cognitive decline. However, CSF markers are significantly more invasive compared to clinical scales or questionnaires; therefore, warranting the need for a predictive model composed of readily available, non-invasive clinical markers. The clinical variables we assessed as predictive factors included neuropsychiatric measures, which were not included as clinical variables in the Schrag et al. study (2017), hence why we did not find hyposmia, RBD or depression as factors that predict cognitive decline. The neuropsychiatric measures, in our study, had a stronger predictive power for CI than UPSIT, GDS and RBDQ scores, as reported by Schrag and colleagues (Schrag et al. 2017).

Older age is a well-recognized risk factor for cognitive decline in both PD and the general population (Williams-Gray et al. 2013). Our findings are in line with this, as age at onset above 60 years served as one of the strongest predictors for CI development for both Level 1 and Level 2 criteria. Furthermore, we also found that higher baseline motor symptom severity, as measured with MDS-UPDRS Part-III, was associated with cognitive decline, defined by Level 1 criteria, which has been previously reported to predict dementia over a 10-year period (Williams-Gray et al. 2013). Although several risk models have been developed to aid the prediction of dementia in Alzheimer’s disease, there is no validated, clinically available too to predictive early drug-naïve PD patients at higher risk of CI. To note, our models, which incorporated several clinical variables, including age at onset, strengthened the power of prediction and the accuracy of cognitive decline compared to single clinical variables alone. Moreover, in our study, when baseline clinical measures were recorded, PD patients had < 24 months disease duration, therefore, potentially offering clinicians with a more accurate understanding of disease prognosis from the easiest stages of the disease. The ability to identify those at risk of cognitive decline in PD at this stage is critical for the early intervention and patient management strategies. Importantly, the current study highlighted the combination of three measures, which are easy to collect within a clinical setting without high associated costs. While the model based on Level 1 diagnostic criteria gives higher predict risk score, of 87% compared to 64% for the Level 2 model, the Level 2 diagnostic criteria gives a higher level of diagnostic certainty (Litvan et al. 2012). Therefore, the predictive model based on the Level 2 diagnostic criteria could offer a more robust model to identify early drug-naïve PD patients at risk to develop CI. Moreover, this model could reflect a characteristic profile of early PD at higher risk of cognitive decline.

The implication of our study supports prognostic and management choices in clinical practice and may aid in optimising treatment options. A thorough evaluation of a patients’ cognitive status is considered prior to a decision for deep brain stimulation (DBS); moreover, having reliable models and algorithms to help predict patients at higher risk to develop cognitive impairment could be an important tool for further stratification and identification of patients for DBS (Pollak 2013). Currently, dopamine replacement therapy (DRT) with standard and advanced treatments provides good options for managing motor symptomatology. Gene- and cell-based therapies offer a potentially viable disease-modifying treatment option in PD, with the potential to provide long-term benefit that is superior to that achieved with DRT as well as expanding beyond the nigrostriatal system (Axelsen and Woldbye 2018; Barker and Transeuro Consortium 2019). However, selecting patient subgroups who are most likely to benefit from these treatment options, with minimal side-effects is imperative for their management. Considering that non-dopaminergic mechanisms are likely to contribute to the disease course in PD patients who undergo cognitive decline in PD (Schulz et al. 2018), this subset of PD patients may be less likely to benefit from dopaminergic and perhaps non-oral therapies. Acetylcholinesterase inhibitors, which have been proven to be beneficial in PD patients with dementia (Pagano et al. 2015), may also benefit patients who are at higher risk of cognitive decline. Early intervention with acetylcholinesterase inhibitors, which could be as early as when the diagnosis is made, could potentially improve disease progression and patient care. Studies evaluating the efficacy of acetylcholinesterase inhibitors, as well as other potential therapies for PD dementia, should be performed in PD patients that are at higher risk of developing CI, who can be identified using our models.

The findings of this study may also aid the selection process of PD patients for future clinical trials. There is significant heterogeneity in CI definition, which could ultimately lead to the failure of clinical trials, as enrolled patients may not be well-characterised for the study. Disease-modifying trials researching into preventing or reversing CI may implement disparate definition of cognitive decline as their endpoint, so would, therefore, need to ensure the correct patients are enrolled in their study. Here, we demonstrate that depending on the definition of CI, based on either Level 1 or Level 2 diagnostic criteria, different models composed of specific clinical assessments can predict cognitive decline. Therefore, highlighting the importance to take into consideration the definition of CI for the specific predictive model used in future clinical trials looking to intervene in CI pathophysiology in PD. Furthermore, considering the implications of patient selection for clinical trials, it is important to note that follow-up evaluations, for two case reports, of European foetal cell transplantation trials conducted in the 1990s concluded that better outcomes were related to receiving treatment earlier in the disease course and subsequently in patients with initial better motor ability (Kefalopoulou et al. 2014). Indeed, younger age (less than 65 years) patients, with shorter disease duration and lower H&Y score are included in the ongoing TransEuro trial of dopaminergic cell replacement therapy in PD patients (Barker and Transeuro Consortium 2019). Although long-term results from this trial are not yet available, it is indicative that optimal group characteristics are of importance in the success of clinical trials.

The limitation of this study is the grouping process. This method is rather stringent in terms of inclusion characteristics, which has invariably led to a restricted number of patients in each cohort. This is reflected in Figs. 1 and 2, where a small number of patients constitute each group after stratifying for each variable. This inevitably influenced the given risk, but also highlights that this study is highly exploratory. However, the advantage of this grouping method is that we could control the variables hypothesised to be predictive of cognitive decline. Furthermore, this study demonstrates the feasibility of using clinically available, easy-to-administer and non-invasive assessments to identify drug-naïve PD patients who are at risk of developing CI, particularly within the clinical setting. Our model for prediction of CI in PD should, however, be further validated with a larger cohort of drug-naïve PD patients with a longer follow-up period, to permit a correct interpretation in clinical settings. Moreover, additional factors should be considered for future models which could contribute towards clinical outcomes and the prediction of cognitive decline including different genetic profiles which have been shown to play a role in the variability of cognitive outcomes and rates of decline (Fagan and Pilstrom 2017; Liu et al. 2017). The inclusion of genetic profiles, into future predictive models of cognitive decline, could be important to facilitate the accurate stratification of PD patients for clinical trials striving towards tailored personalised therapeutic approaches.

## Conclusions

In conclusion, we demonstrate that clinically available measures, including age at onset > 60 years, MDS-UPDRS III score > 20 and Semantic Fluency, Symbol Digit or Hopkin’s Verbal Test scores > 35 are most likely to predict a poor cognitive outcome early in the disease course. Considering the ongoing development of new treatment options for PD, the characterisation of those patients most likely to benefit from early intervention and inclusion in clinical trials for cognitive decline is crucial.

## Data Availability

Data used in the preparation of this article were obtained from the Parkinson’s Progression Markers Initiative (PPMI) database (www.ppmi-info.org/data). For up-to-date information on the study, visit www.ppmi-info.org. PPMI data are available to the research community on the PPMI website as it is collected. All PPMI standardized protocols and data are available at www.ppmi-info.org.

## Abbreviations

PD: Parkinson’s disease
CI: Cognitive impairment
HR: Hazard ratio
MoCA: Montreal cognitive assessment
MDS-UPDRS: Movement Disorder Society sponsored Unified Parkinson Disease Rating Scale
H&Y: Hoehn and Yahr
