# Correction to: Predict cognitive decline with clinical markers in Parkinson’s disease (PRECODE-1)

**DOI:** 10.1007/s00702-022-02476-7

**Published:** 2022-03-01

**Authors:** Heather Wilson, Gennaro Pagano, Tayyabah Yousaf, Sotirios Polychronis, Rosa De Micco, Beniamino Giordano, Flavia Niccolini, Marios Politis

**Affiliations:** Neurodegeneration Imaging Group, Maurice Wohl Clinical Neuroscience Institute, Institute of Psychiatry, Psychology and Neuroscience (IoPPN), King’s College London, 125 Coldharbour Lane, Camberwell, London, SE5 9NU UK

## Correction to: Journal of Neural Transmission (2020) 127:51–59 10.1007/s00702-019-02125-6

The original version of this article unfortunately contained a mistake. The following article note is missing. Gennaro Pagano is joint first author with Heather Wilson.

